# Integrating network pharmacology, molecular docking and experimental verification to explore the therapeutic effect and potential mechanism of nomilin against triple-negative breast cancer

**DOI:** 10.1186/s10020-024-00928-2

**Published:** 2024-09-28

**Authors:** Zhixuan Wu, Haoyi Xiang, Xiaowu Wang, Rongrong Zhang, Yangyang Guo, Liangchen Qu, Jingyao Zhou, Yanyi Xiao

**Affiliations:** 1 The Dingli Clinical College of Wenzhou Medical University, Wenzhou, Zhejiang Province 325000 China; 2 Zhejiang Key Laboratory of Intelligent Cancer Biomarker Discovery and Translation, First Affiliated Hospital, Wenzhou Medical University, Wenzhou, Zhejiang Province 325035 China; 3 Zhejiang University School of Medicine, Hangzhou, Zhejiang Province 310016 China; 4 Department of Colorectal Surgery, Sir Run Run Shaw Hospital of Zhejiang University, Hangzhou, Zhejiang Province 310016 China; 5 Department of Burns and Skin Repair Surgery, The Third Affiliated Hospital of Wenzhou Medical University, Ruian, 325200 China; 6 Emergency Department, Taizhou Hospital of Zhejiang Province Affiliated to Wenzhou Medical University, Taizhou, 318000 China; 7 Pharmacy Department, Taizhou Central Hospital, Taizhou, Zhejiang Province 318000 China; 8 Department of Thyroid and Breast Surgery, Wenzhou Central Hospital, The Second Affiliated Hospital of Shanghai University, Wenzhou, Zhejiang Province 325000 China

**Keywords:** Network pharmacology, Molecular docking, Nomilin, Triple negative breast cancer, PI3K/Akt pathway

## Abstract

**Background:**

Nomilin is a limonoid compound known for its multiple biological activities, but its role in triple negative breast cancer (TNBC) remains unclear. This study aims to uncover the potential therapeutic effect of nomilin on TNBC and elucidate the specific mechanism of its action.

**Methods:**

We employed weighted gene co-expression network analysis (WGCNA), differential expression analysis, and the GeneCards database to identify potential targets for TNBC. Simultaneously, we utilized the Swiss Target Prediction, ChEMBL, and STITCH databases to identify potential targets of nomilin. The core targets and mechanisms of nomilin against TNBC were predicted through protein-protein interaction (PPI) network analysis, molecular docking, and enrichment analysis. The results of the network pharmacology were corroborated by conducting experiments.

**Results:**

A total of 17,204 TNBC targets were screened, and 301 potential targets of nomilin were identified. Through the PPI network, eight core targets of nomilin against TNBC were pinpointed, namely BCL2, Caspase3, CyclinD1, EGFR, HSP90AA1, KRAS, PARP1, and TNF. Molecular docking, molecular dynamics simulation and proteome microarray revealed that nomilin exhibits strong binding activity to these core proteins. Enrichment analysis results indicated that the anti-TNBC effect of nomilin is associated with PI3K/Akt pathway. In vitro and in vivo experiments have demonstrated that nomilin inhibits TNBC cell proliferation and migration while promoting cell apoptosis through the PI3K/Akt pathway.

**Conclusion:**

For the first time, the research effectively discovered the objectives and mechanisms of nomilin in combating TNBC using network pharmacology, molecular docking, molecular dynamics simulation, proteome microarray and experimental confirmation, presenting a hopeful approach for treating TNBC.

**Graphical Abstract:**

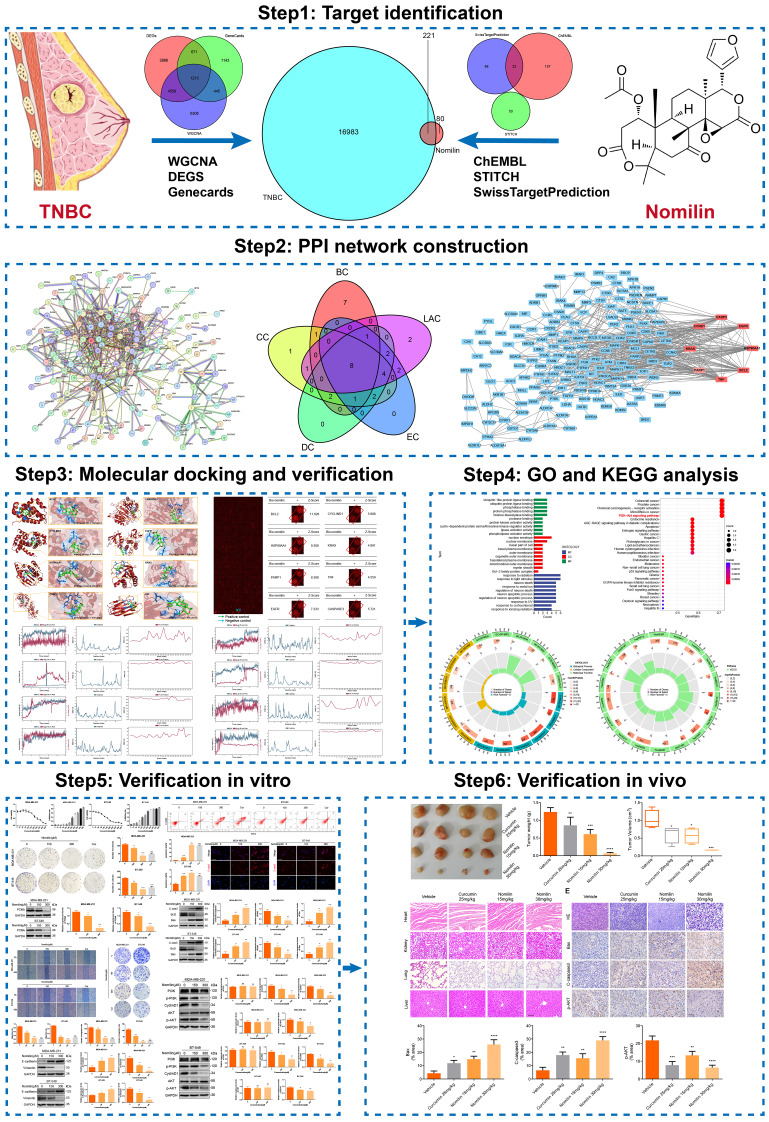

**Supplementary Information:**

The online version contains supplementary material available at 10.1186/s10020-024-00928-2.

## Introduction

In 2020, the International Agency for Research on Cancer reported a significant increase in the incidence of breast cancer (BRCA), solidifying its status as the most common type of cancer worldwide (Sung et al. 2021; Liao et al. 2023). Furthermore, BRCA is the second most common cause of cancer-related mortality among women (Sun et al. 2017; Li and Cheng, Yun, Cheng, Junchi, Yao, Jincao, Song, Mei 2023; Yan et al. 2023). Triple negative breast cancer (TNBC) is a form of BRCA that is distinguished by the lack of estrogen receptors (ER), progesterone receptors (PR), and human epidermal growth factor receptor 2 (HER2) (Jiang et al. 2019; Chen et al. 2023). This type of breast cancer is associated with three key characteristics: occurrence at a younger age, a high recurrence rate, and strong invasiveness (Yan et al. 2023). This particular form of breast cancer is widely regarded as one of the most hostile. Due to the insufficient expression of ER, PR and HER2 in TNBC tumors, patients derive limited benefit from existing endocrine and targeted therapies (Derakhshan and Reis-Filho 2022). As a result, chemotherapy remains the primary systemic treatment option. While TNBC has shown relative sensitivity to anthracyclines and taxanes (Bai et al. 2021), their application is limited by serious side effects and drug resistance. Natural products are known for their favorable therapeutic effects, high safety profile, minimal side effects, and broad potential, making them a promising option for clinical tumor treatment (Chen et al. 2019). Therefore, the development of new natural products as complementary and alternative therapies for TNBC is of utmost importance.

Extracted from citrus fruits, nomilin is a limonoid compound that exhibits various biological effects such as anti-inflammatory, anti-obesity, antioxidant, anti-osteoclastogenesis, neuroprotection, immune regulation, and detoxification (Raphael and Kuttan 2003; Kimira et al. 2015; Shi et al. 2019). Furthermore, a growing body of research has shown the noteworthy anti-cancer efficacy of nomilin. It has been found to suppress the proliferation of pancreatic cancer and induce apoptosis by cleaving Caspase3 (Chidambara Murthy et al. 2021). Additionally, there have been reports of nomilin demonstrating toxic effects on neuroblastoma and colon adenocarcinoma (Poulose et al. 2006). The research conducted by Lam et al. provided evidence that nomilin effectively suppressed the development of benzo(α)pyrene-induced forestomach tumors in mice (Lam and Hasegawa 1989). Nomilin was also found to impede tumor cell invasion and the formation of metastatic lung tumor nodules (Pratheeshkumar et al. 2012), while also showing potential as an inhibitor of tumor angiogenesis (Pratheeshkumar and Kuttan 2011). However, the precise effects of nomilin on the proliferation, viability, and metastasis of TNBC remain incompletely elucidated. Additional investigations are warranted to delineate and elucidate the specific effects that nomilin may have on this particular type of BRCA.

The complex array of targets influenced by nomilin poses difficulties in fully elucidating its pharmacological mechanism through conventional means. Network pharmacology is a burgeoning interdisciplinary field that combines systems biology and bioinformatics, providing notable benefits in the identification of drug targets and the elucidation of intricate drug mechanisms (Zeng et al. 2022; CHEN 2023; LV et al. 2022, 2023; Shahrajabian et al. 2023). Accordingly, this research employs network pharmacology to elucidate the potential anti-tumor mechanism of nomilin at a systemic level, facilitating the identification of multiple drug targets and enhancing understanding of its therapeutic effects. Additionally, the study explored the binding mode and affinity between nomilin and potential targets through molecular docking, and ultimately conducted preliminary validation through cell and animal experiments. The workflow of the study is visually summarized in Graphical abstract.

## Materials and methods

### Data sources and processing

The TNBC RNA-seq data was acquired from The Cancer Genome Atlas (TCGA) data portal, and the transcription profiles were downloaded as fragments per kilobase (FPKM) for further analysis. The “limma” R package was used to identify differentially expressed genes (DEGs) in the TNBC cohort by comparing paracancerous tissue and tumor tissue. A screening threshold of |log2FC| > 1 and adjusted *P* < 0.05 was applied.

### Weighted gene co-expression network analysis (WGCNA)

The TCGA-TNBC expression profile was utilized as the input dataset for WGCNA. The goodSamplesGenes method within the “WGCNA” R package was utilized initially to remove outlier genes and samples. First, genes with small fluctuations in the dataset were removed based on the median absolute deviation (MAD), and the remaining gene expression files were entered into WGCNA; second, the pick-Soft-Threshold function was used to calculate the adjacency using the soft threshold power b obtained from the co-expression similarity. Third, the adjacency relationships were converted into a topological overlap matrix (TOM) and the respective dissimilarity (1-TOM) was evaluated. Fourth, hierarchical clustering combined with a dynamic tree cut function was used to identify modules. In order to classify genes with similar expression profiles into gene modules, average linkage hierarchical clustering was used based on the TOM-based dissimilarity measure, and the minimum size of the gene tree diagram (genomes) was 50. Then, for the modules most closely associated with TNBC, the module membership as well as the gene significance were calculated. Finally, the characteristic gene network was visualized.

### Identification of potential targets for TNBC

Potential targets related to TNBC were screened from the GeneCards database by searching the keyword “triple-negative breast cancer”. The genes obtained from DEGs, WGCNA, and those screened from the GeneCards database were used as input sets to create a venn diagram.

### Identification of potential anti-TNBC targets for nomilin

The molecular structure of nomilin was shown in Supplementary Fig. 1A-B. Nomilin’s potential targets were screened from the Swiss Target Prediction database, the medicinal chemistry database ChEMBL, and the STITCH database. In general, we first obtained the SMILES of nomilin from the PubChem website and then put it into the Swiss Target Prediction database. In addition, potential targets related to nomilin were screened from the medicinal chemistry database ChEMBL, and the STITCH database by searching the keyword “nomilin”. After summarizing the Uniprot IDs, removing duplicates, and drawing a venn diagram, 301 potential targets of nomilin were identified. A venn diagram was also used to analyze the overlap between nomilin’s potential targets and TNBC targets, resulting in the identification of 221 potential anti-TNBC targets for nomilin. These 221 potential anti-TNBC targets of nomilin were then imported into Cytoscape software to construct a “drug-target-disease” network.

### Constructing a protein-protein interaction (PPI) network for the shared targets of nomilin and TNBC

To obtain PPI information, the interactive genetic database search tool String was utilized. The PPI network was constructed by inputting 221 potential targets of nomilin against TNBC into the String database, selecting the Homo sapiens species, setting the minimum interaction score to high confidence (0.700), and removing disconnected nodes in the network.

### Core target identification of nomilin for TNBC

In order to ascertain the principal targets of nomilin against TNBC, the PPI network was integrated into Cytoscape, and the CytoNCA plug-in was employed to conduct an analysis of betweenness centrality (BC), closeness centrality (CC), degree (DC), eigenvector centrality (EC), and local average connectivity (LAC) parameters for the relevant targets (Shi et al. 2021). By selecting the top 20 genes with the most elevated scores in every analysis and utilizing a venn diagram, 8 core targets were identified due to the presence of overlapping genes.

### Computer-aided molecular docking

Protein structures of the eight main targets were obtained from the PDB database (Bcl-2 ID:1K3K; Caspase-3 ID:1CP3; CCND1 ID:6P8E; EGFR ID:2XKN; HSP90AA1 ID:5NJX; KRAS ID:6N65; PARP1 ID:7KK2; TNF ID:1CA4). Proteins were purified by eliminating duplicate conformations and water molecules, and the protein pockets’ binding sites were forecasted. Original ligands were then deleted. The results were input into AutoDock, and Autodock Tools were used to dehydrate and hydrogenate the proteins and drug molecules in the computer. Afterwards, molecular docking was performed, the optimal docking conformation was chosen based on low-energy and reasonable conformation principles, set the docking site to Ligand Atoms, and set the amino acids within 45° from the ligand molecule as the docking pocket, and the obtained results were saved along with the recorded affinity values. Stable binding between the ligand and the receptor molecule is indicated by a negative binding energy, with greater absolute values representing increased stability. Subsequently, the outcomes were imported into PyMOL for additional visualization.

### Molecular dynamics simulation

The simulation was conducted utilizing the Schrodinger 2019 software module “Desmond”. The predefined simple point-charge (SPC) water model was employed, along with the OPLS2005 force field, to simulate water molecules. To neutralize the system charge, chloride ions/sodium ions were randomly added in appropriate amounts to balance the charge within the solvation system. Energy minimization is conducted utilizing the standard protocol incorporated within the Desmond module. Following this, a normal pressure and temperature (NPT) simulation was carried out for a duration of 100 ns, with data being recorded at intervals of 50 ps. The progress of the simulation was tracked and saved accordingly.

### Proteome microarray assay

The HuProt™ 20 K Human Proteome Microarrays from CDI Laboratories, Inc, were employed for chip hybridization, washing, and detection following standard chip detection procedures. In a nutshell, the chip was immersed in a 5% BSA blocking solution and incubated with biotin (10 µM) and biotin-nomilin (10 µM) for 1 h. After thorough washing, the chip was placed in a 0.1% Cy5-Streptavidin Solution (Cy5-SA) and allowed to react for 20 min in the dark. Following further washing and centrifugation, the microarray was scanned at 635 nm using the GenePix 4000B scanner. The median (M) and standard deviation (SD) of the raw signal intensities (I) from all sites were calculated to determine the Z-Score of the corrected data for each site. Z-Score = (I-M) / SD.

### Enrichment analysis

The 8 core targets were analyzed using R software to identify the biological processes and pathways through Gene Ontology (GO) and Kyoto Encyclopedia of Genes and Genomes (KEGG) analysis via the R “ClusterProfiler” package, P < 0.05 were regarded as significant. The above results were visualized by R“ggplot2” and “enrichplot” packages eventually. Histograms were used to visualize the initial 10 entries of GO analysis, encompassing cellular component, molecular function (MF), and biological process (BP), whereas bubble charts were employed to represent the first 30 entries of KEGG analysis results. Afterwards, the R programming language was employed to produce circular diagrams illustrating the main signaling pathways discovered in the GO and KEGG examinations.

### Reagents and cell culture

Nomilin (purity 99.38%), the compound with the CAS number 1063-77-0 was bought from MedChemExpress (MCE, NJ, USA). Curcumin (CAS number 458-37-7) with a purity of 98.16%, obtained from MCE, was utilized as a positive control. The MDA-MB-231 and BT-549 cell lines, which are human TNBC cell lines (TNBCCs), were acquired from the Shanghai Institute of Cell Biology, Chinese Academy of Sciences. TNBCCs were cultured in DMEM medium and RPMI-1640 medium, respectively. In order to augment cellular culture conditions, a mixture of 10% FBS and 1% penicillin-streptomycin was incorporated into all media. The cells were subsequently incubated in a humidified environment with 5% CO2.

### Cell counting Kit-8 (CCK-8) assay

TNBCCs were placed in 96-well plates with a cell density of 0.8 × 10^4^ cells per well. After exposing the cells to different amounts of nomilin (0, 10, 20, 50, 100, 150, 200, 250, 300, 400, 500 µM) for a duration of 24 h, 10 µL of cck-8 reagent (Targetmol, Shanghai, China) was introduced into each well and left to incubate for 4 hours. The absorbance of each well was then measured at 450 nm.

### Colony formation

TNBCCs were placed in 6-well plates with a concentration of 1000 cells per well. After the formation of visible cell clumps, they were incubated with 0, 150, and 300 µM nomilin for 24 h. After counting approximately 100 cells in the control group’s single-cell colony, the cells were fixed using 4% paraformaldehyde, stained with crystal violet for a duration of 15 min, air-dried, and subsequently photographed and enumerated.

### Western blot (WB)

WB analysis was conducted as described previously (Wu et al. 2023). In summary, cells were disrupted using RIPA solution, and the overall protein concentration was assessed utilizing the BCA technique. The denatured total protein was then subjected to polyacrylamide gel electrophoresis. To remove protein bands of comparable molecular weights, we employed a stripping buffer (Beyotime Biotechnology, Shanghai, China) for membrane stripping. Supplementary Table 1 contains a list of the main antibodies utilized. Quantitative analysis of the images was carried out using Image J software.

### Wound healing assay

TNBCCs were placed in a six-well dish until they developed a complete layer of cells. To create a linear gap on each plate, a 1000 µl pipette tip was used to scratch once. After rinsing the isolated cells with PBS, a serum-free solution was introduced, which included nomilin at concentrations of 0, 150, or 300 µM. Following a 2-day period, the culture area was examined under an inverted microscope to capture images.

### Transwell assay

Dispense 200 µl of serum-free medium containing 8 × 10^4^ cells into the upper chamber, and add 500 µl of medium with 20% FBS to the lower chamber. After cells have adhered, replace the medium in the upper chamber with serum-free medium containing 0, 150, or 300 µM nomilin. Subsequently, fix and stain the cells with crystal violet. Utilize a microscope to tally the migrating cells in various regions of the slide.

### Flow Cytometry

Following a 24-hour incubation period of TNBCCs with nomilin, the cells were subjected to trypsin digestion and subsequent centrifugation. Following three washes with PBS, the cells were resuspended in a buffer solution. Next, the reconstituted cells were cultured with 5 µl FITC for a duration of 15–20 min. Following this, 5 µl 7-AAD was added for 5 min in order to conduct apoptosis analysis. The BD FACSVerse™ system was utilized to examine the apoptosis of TNBCCs through flow cytometry.

### Immunofluorescence (IF)

Following a 24-hour treatment of TNBC cells with nomilin, the cells were then fixed and permeabilized using 0.1% Triton X-100. After that, the cells were obstructed using goat serum for a duration of 30 min and then subjected to the Cleaved Caspase3 primary antibody, which was left to incubate overnight at 4 °C. Afterward, the cells underwent three rounds of washing with PBS and were subsequently exposed to secondary antibodies at 37 °C for 1 h. Afterward, the cells were incubated with DAPI for 7 min, and finally mounted with an anti-fade fluorescence mounting medium. Fluorescence microscopy was utilized to capture IF images of the cells.

### Xenograft tumor experiments

A group of sixteen BALB/c mice, all female and without fur, weighing between 18 and 22 g and aged 6 to 8 weeks, were housed in a controlled environment with regulated temperature, humidity, and lighting. They were provided with standard nourishment and access to water. Four groups were formed by randomly dividing mice. Subsequently, a total of 5 × 10^6^ MDA-MB-231 cells were subcutaneously implanted into the right thigh root of mice. The mice in the nomilin group were administered nomilin at a dose of 15/30 mg/kg·2d (ip). The control group mice received injections of DMSO as a solvent whereas mice in positive control group were treated with curcumin (25 mg/kg·2d). Carbon dioxide asphyxiation was used to euthanize all mice, and tissues from the tumor, heart, liver, lung, and kidney were gathered for further experiments. The Institutional Animal Care and Utilization Committee of Wenzhou Medical University granted approval for this study involving animals on December 7, 2022 (WYYY-AEC-2022-062). Moreover, animal tests were conducted in compliance with the regulations set by all animal welfare governing bodies(Kilkenny et al. 2010).

### Hematoxylin-eosin (HE) staining and immunohistochemistry (IHC) staining

The tumor and organ samples were preserved in formalin, encased in paraffin, and subsequently sliced into sections that were 4 μm in thickness. HE was used to stain both the organs and tumor tissues. IHC analysis was conducted following established protocols (Dai et al. 2018). The tumor tissue sections underwent deparaffinization using xylene, followed by rehydration and antigen retrieval, prior to incubation with primary antibodies. Subsequently, the sections were treated with enzyme-labeled secondary antibodies and diaminobenzidine, and finally analyzed.

### Statistical analysis

In the field of network pharmacology, all statistical analyses were conducted using R software. Experimental data were analyzed using GraphPad Prism for statistical purposes. The data results were subjected to one-way analysis of variance, with statistical significance defined as *p* < 0.05. To ensure reliability, each independent experiment was conducted a minimum of three times. Comparative analyses between two groups were executed using Student’s t-test, whereas one-way ANOV A was employed for comparisons involving multiple groups. p-value < 0.05 was considered to indicate statistical significance.

## Results

### Acquisition of potential targets of nomilin for the treatment of TNBC

**Fig. 1:**
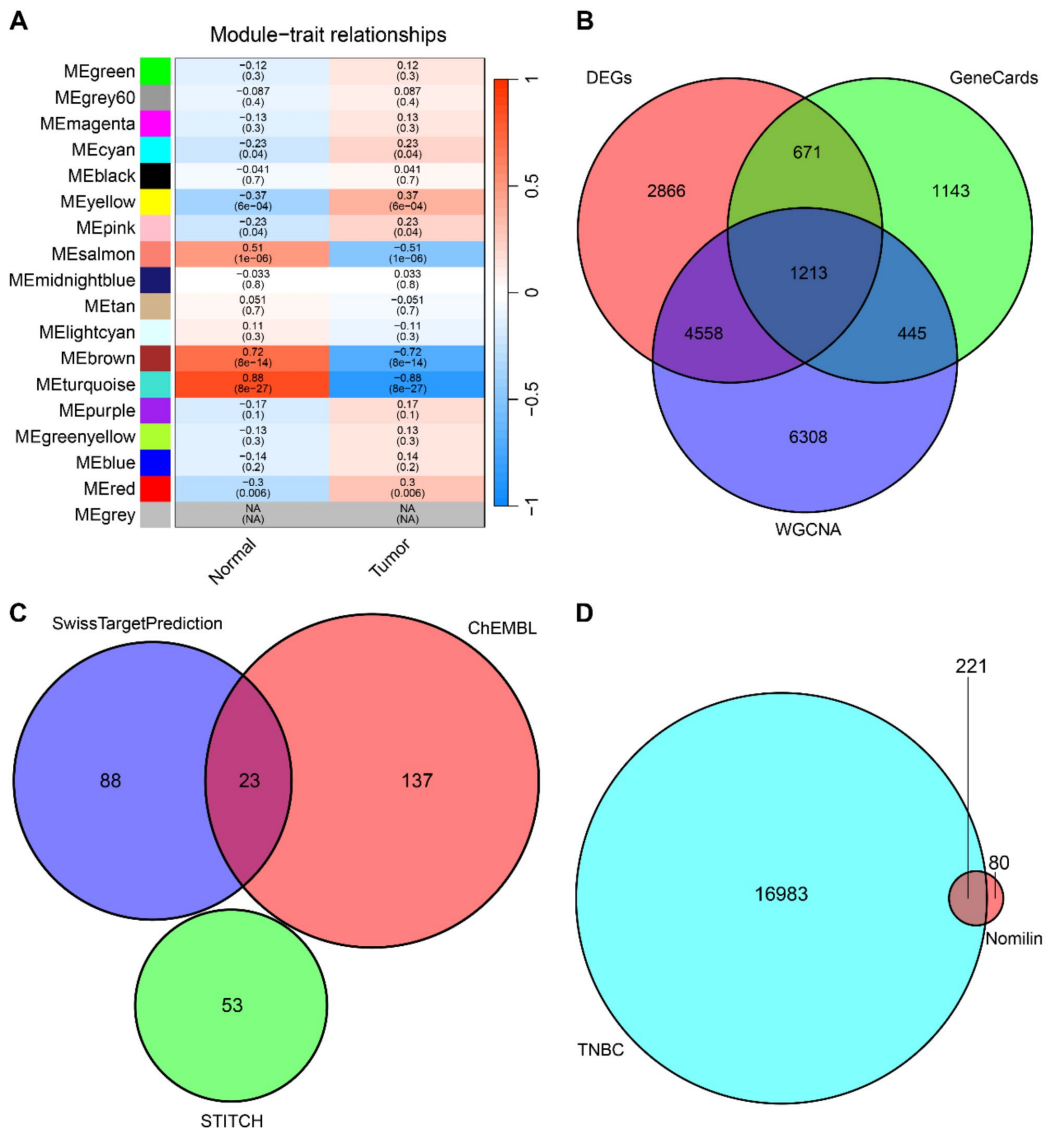
Identification of potential targets of nomilin against TNBC. (**A**) Identification of the module most strongly associated with TNBC phenotype by WGCNA analysis. (**B**) Venn diagram of targets associated with TNBC. (**C**) Venn diagram of targets of nomilin. (**D**) Creating a venn diagram to illustrate the overlap between targets of nomilin and targets associated with TNBC

**Fig. 2:**
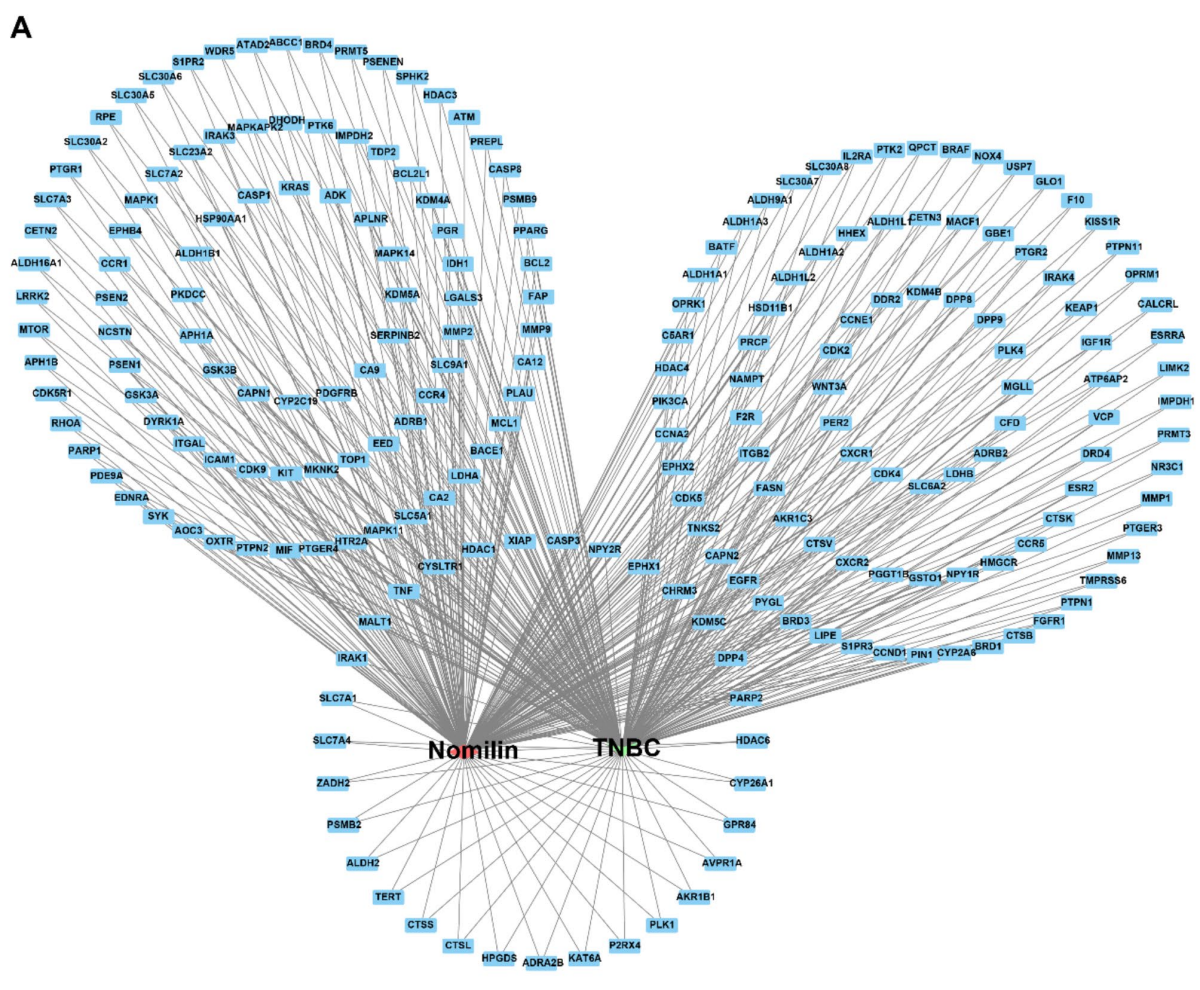
(**A**) The drug-target-disease network of nomilin against TNBC

First, we conducted a differential expression analysis on tumors and adjacent tissues in the TNBC cohort, yielding a total of 9308 DEGs. Subsequently, WGCNA analysis pinpointed specific modules of TNBC tumor characteristics, with the MEturquoise module showing the highest correlation with TNBC tumor phenotype and 12,524 genes extracted from this module (Fig. 1A). We then screened 3472 potential targets related to TNBC from the GeneCards database. Combining the potential targets obtained from the three analysis methods into a venn diagram (Fig. 1B) yielded a total of 17,204 potential targets of TNBC. Following this, using Swiss Target Prediction, ChEMBL, and STITCH databases, we identified 301 potential targets of nomilin (Fig. 1C). Cross-analysis of the 301 targets of nomilin with the 17,204 potential TNBC-related targets revealed 221 intersections as potential targets of nomilin against TNBC (Fig. 1D). Additionally, we utilized Cytoscape to construct the drug-target-disease network of nomilin against TNBC (Fig. 2).

#### The establishment of a PPI network and the identification of core targets

**Fig. 3:**
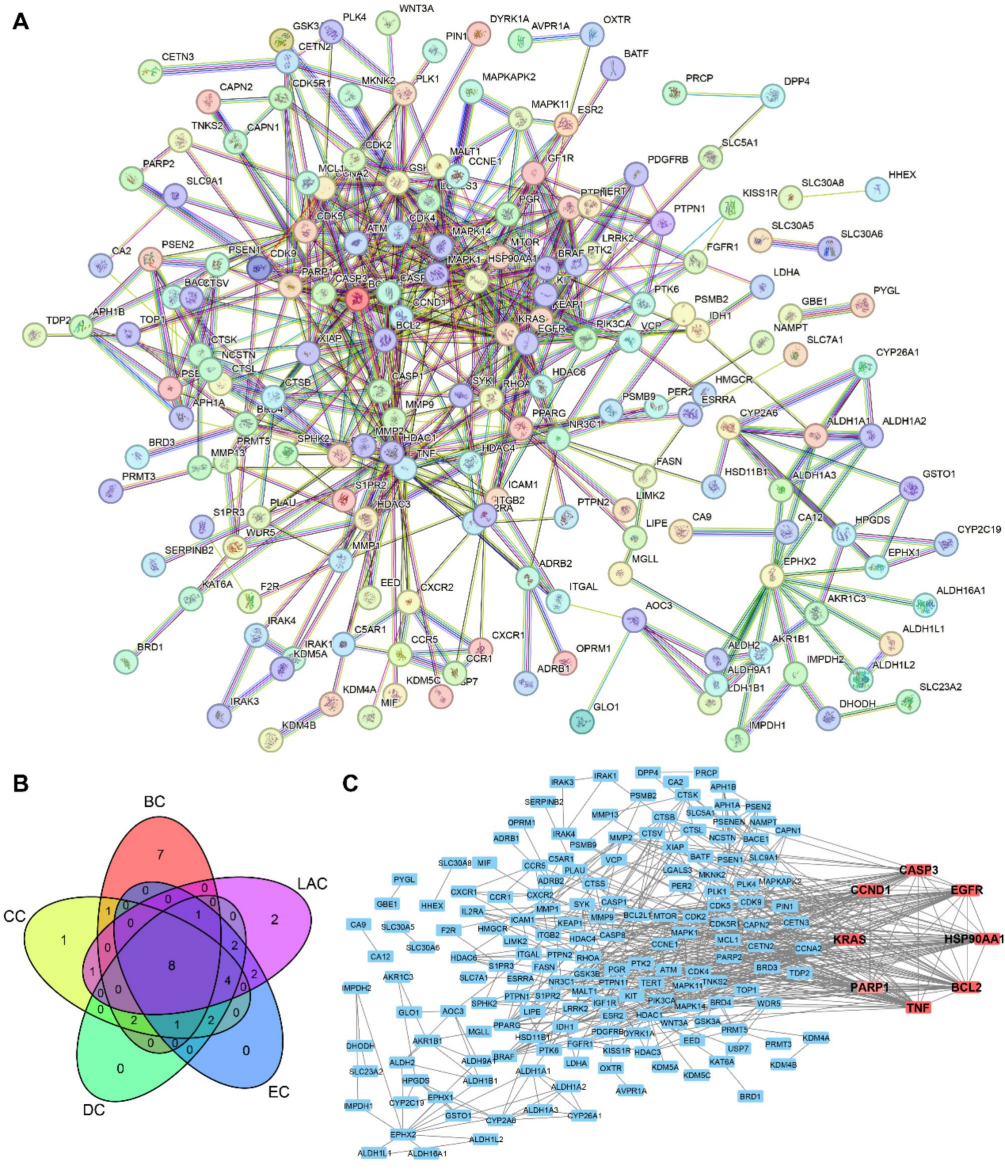
Identification of core targets of nomilin against TNBC. (**A**) A PPI network of the targets of nomilin against TNBC. (**B**) Creating a venn diagram to show the overlap among the top 20 highest scoring genes in the BC, CC, DC, EC, and LAC analyses. (**C**) A PPI network of 8 core targets and other targets

In order to determine the primary focus of nomilin on TNBC, we employed the String database to build a PPI network comprising of 219 nodes and 562 edges (Fig. 3A). Afterwards, we utilized Cytoscape program along with the CytoNCA add-on to perform BC, CC, DC, EC, and LAC examinations. After cross-analyzing the top 20 genes with the most elevated scores from each analysis, a total of 8 core targets were identified: BCL2, Caspase3, CyclinD1, EGFR, HSP90AA1, KRAS, PARP1, and TNF (Fig. 3B). Additionally, Fig. 3C depicts the protein interaction network between the eight core targets and other targets.

#### Molecular docking analysis and dynamics simulation

**Fig. 4:**
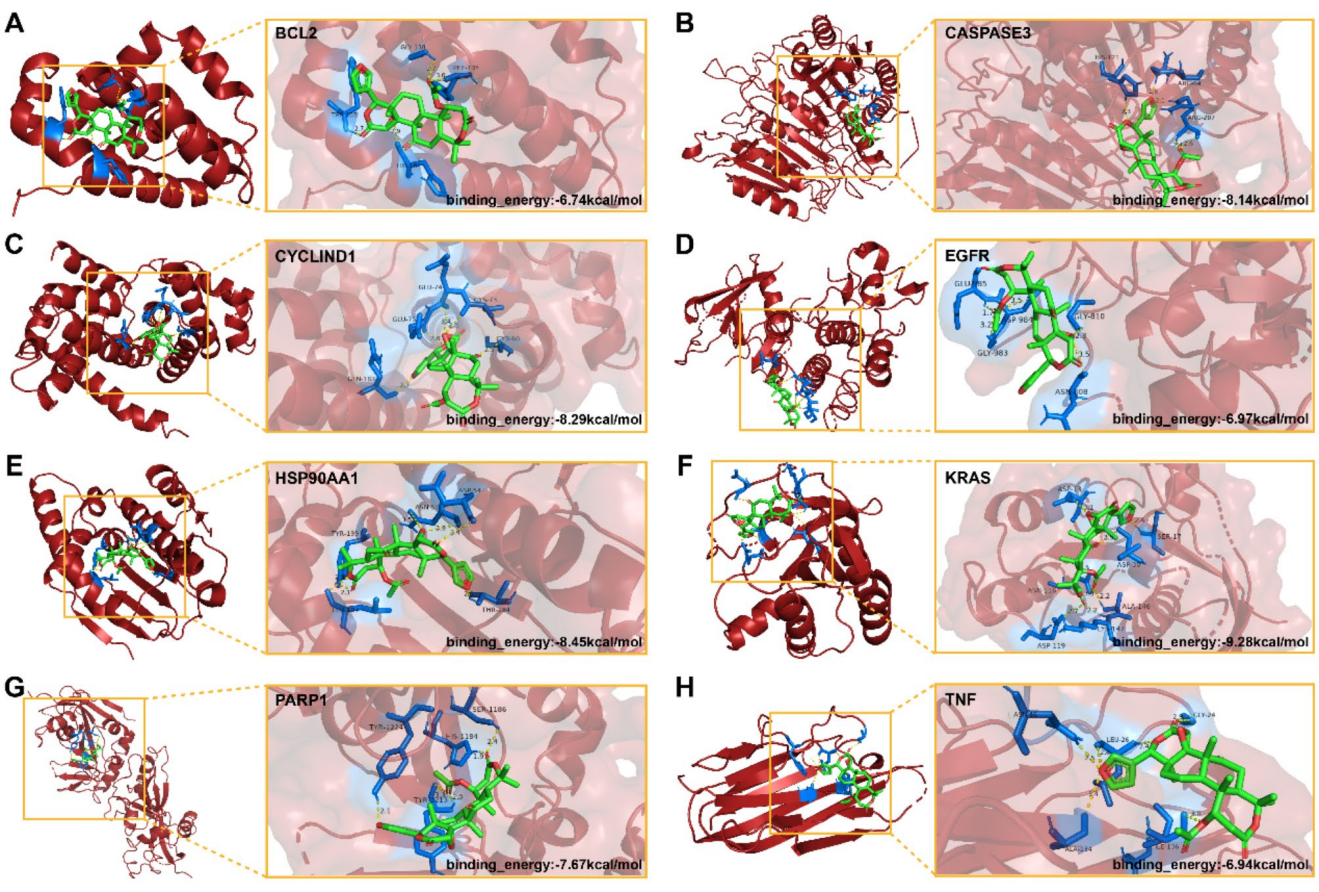
Molecular docking analysis of nomilin and (**A**) BCL2, (**B**) Caspase3, (**C**) CyclinD1, (**D**) EGFR, (**E**) HSP90AA1, (**F**) KRAS, (**G**) PARP1, (**H**) TNF. The yellow lines represent Hydrogen Bond

Subsequently, we conducted molecular docking to assess the potential binding between nomilin and the identified eight core targets. Visualization of the results was accomplished using PyMol software (Fig. 4A-H). The binding energies of nomilin to BCL2, Caspase3, CyclinD1, EGFR, HSP90AA1, KRAS, PARP1, and TNF were determined to be -6.74, -8.14, -8.29, -6.97, -8.45, -9.28, -7.67, and − 6.94 kcal/mol, respectively. Significantly, the results, which were replicated three times, consistently demonstrated that the binding energies of nomilin to all eight core targets were below − 5 kcal/mol (Supplementary Fig. 2). This indicates a favorable binding activity. Furthermore, we conducted molecular dynamics simulations to assess the stability of the binding between nomilin and the core targets. The trajectory of the root mean square deviation (RMSD) revealed that the binding of nomilin to BCL2, CYCLIND1, EGFR, HSP90AA1, PARP1, and TNF remained consistently stable (Supplementary Figs. 3–4). This indicates that nomilin exhibits strong binding affinity towards these targets.

### Proteome microarray analysis of nomilin and core targets

**Fig. 5:**
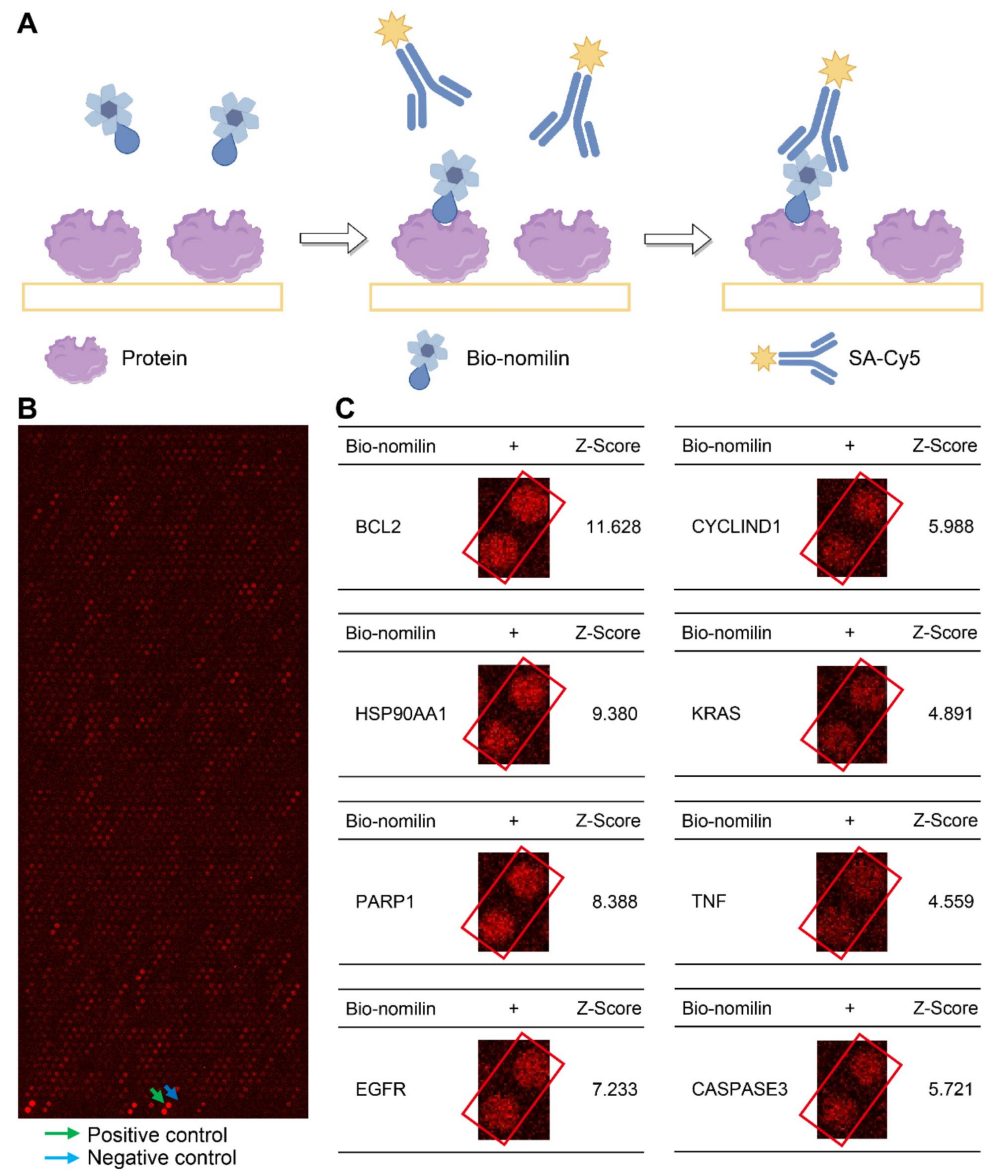
Analysis of the binding activity of nomilin and core targets using a human proteome microarray. (**A**) A schematic representation of the procedure used to detect nomilin-binding proteins utilizing proteome microarrays. (**B**) Human proteome microarrays were probed with Bio-nomilin. Representative images of the protein array revealed a positive control (indicated by a green arrow) and a negative control (indicated by a blue arrow) on a selected area of the microarrays. (**C**) Enlarged images and Z-scores of core targets of the proteome microarray results

The HuProt microarrays, encompassing around 20,000 human full-length proteins, are extensively utilized in small molecule target research (Zhang et al. 2015). In this study, we employed HuProt microarrays to assess the binding affinity of nomilin towards core targets. The microarray was probed with Bio-nomilin, and the resulting binding events were visualized using Cy5-SA (Fig. 5A). Figure 5B displays a subset of the Bio-nomilin microarray results, while Fig. 5C provides enlarged images depicting the interaction between Bio-nomilin and its core targets. Subsequently, Z-Score values were computed for the corrected data at each site. The results indicated a strong association between bio-nomilin and core targets such as BCL2, HSP90AA1, PARP1, EGFR, CYCLIND1, KRAS, TNF, and Caspase3, as evidenced by Z-Scores of 11.628, 9.380, 8.388, 7.223, 5.988, 4.891, 4.559, and 5.721, respectively. This suggests a robust binding affinity between nomilin and its core targets.

### Functional enrichment analysis

**Fig. 6:**
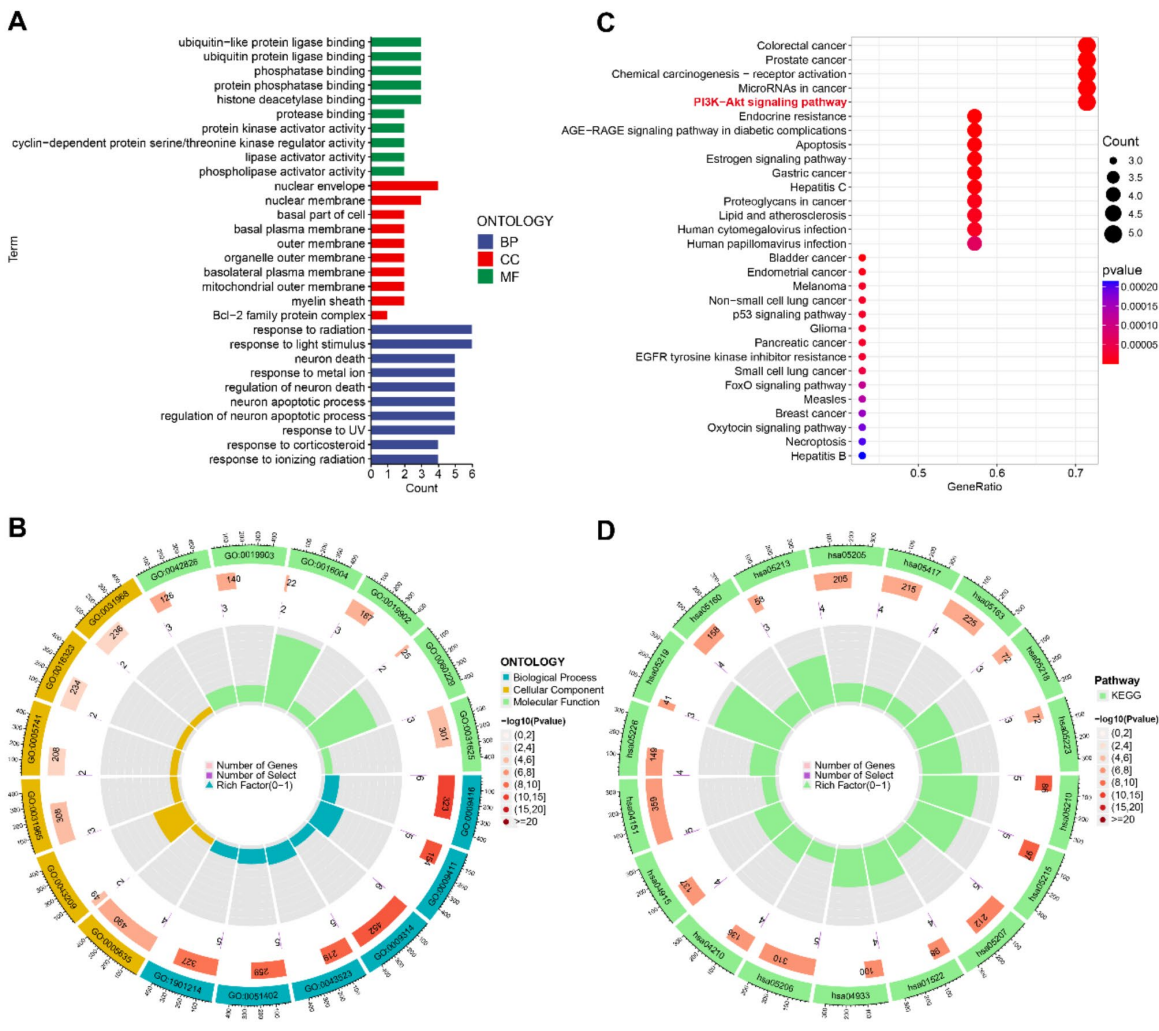
Functional enrichment analysis. (**A**) A histogram of GO enrichment analysis of 8 core targets. (**B**) A circle diagram of GO analysis. (**C**) A bubble chart of KEGG enrichment analysis of 8 core targets. (**D**) A circle diagram of KEGG analysis

In order to elucidate the key biological processes involved in nomilin’s treatment of TNBC, we conducted GO enrichment analysis (Fig. 6A and B). According to the analysis of BP, it was found that the main associations of the eight core targets are with reactions like binding of proteins similar to ubiquitin-like protein ligase, binding of ubiquitin protein ligase and phosphatase. In the meantime, the analysis of cellular component revealed a concentration on reactions associated with the nucleus envelope, nucleus membrane, and the lower section of the cell. Furthermore, the MF analysis highlighted the relevant targets’ involvement in responses to radiation, light stimulus, and neuron death. In order to investigate the signaling pathways linked to the 8 main targets, we conducted KEGG enrichment analysis (Fig. 6C and D). The findings indicated that the signaling pathways closely associated with these central objectives encompass colorectal cancer, prostate cancer, chemical carcinogenesis through receptor activation, microRNAs in cancer, the PI3K-Akt signaling pathway, resistance to endocrine therapy, apoptosis, and the estrogen pathway. The results clearly demonstrate the strong correlation between the anti-TNBC impact of nomilin and these pathways. One of the pathways with the highest number of enriched genes, the PI3K/Akt pathway, may play a significant role in mediating nomilin’s anti-TNBC effect.

## Nomilin inhibits the proliferation of TNBCCs

**Fig. 7:**
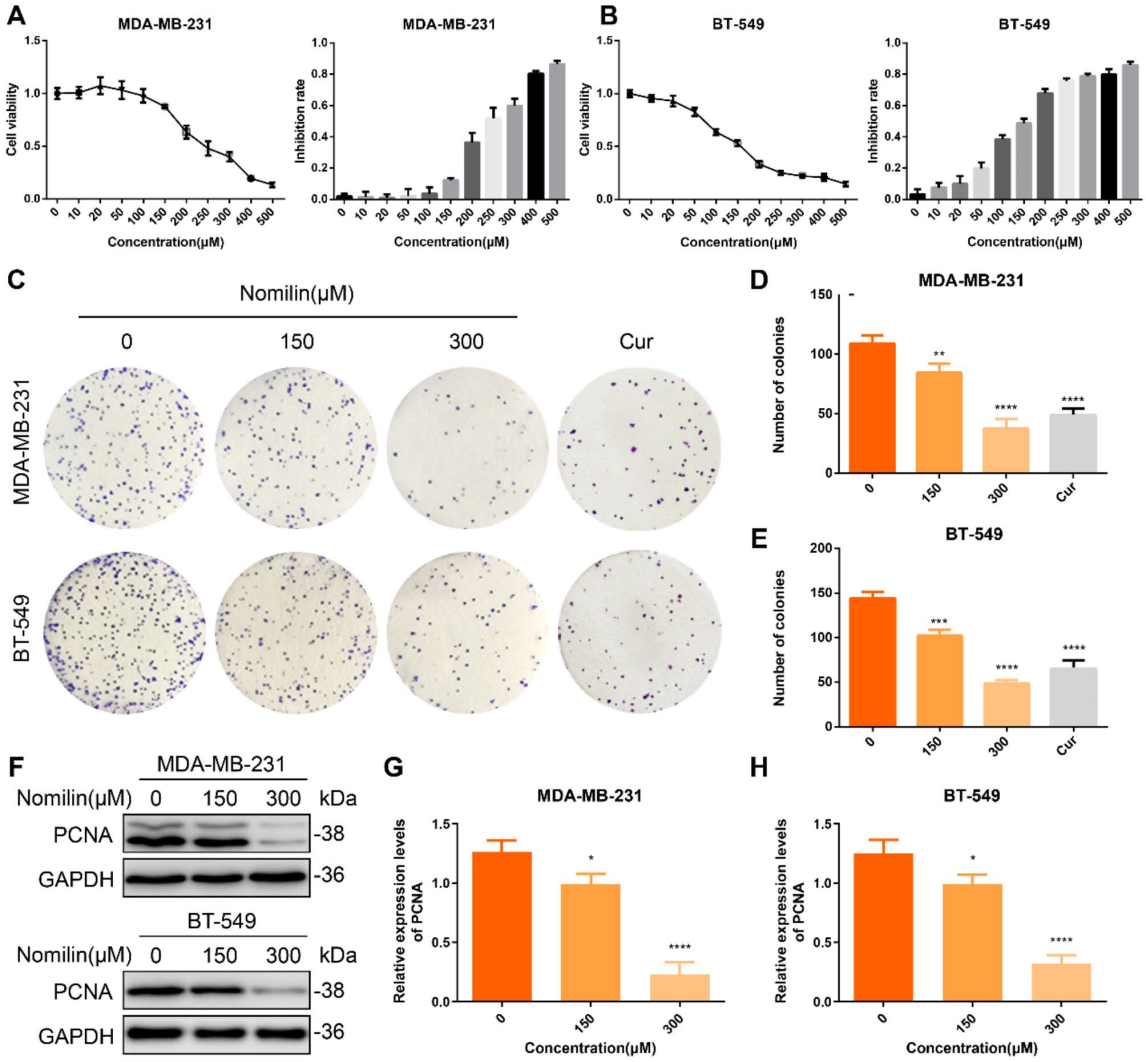
Nomilin inhibits the proliferation of TNBC cells. Viability of (**A**) MDA-MB-231 and (**B**) BT-549 cells with or without nomilin treatment analysed by CCK-8 assay. (**C**-**E**) The colony formation assay was utilized to analyze the proliferation of TNBC cells with or without nomilin. (**F**-**H**) Western blot analysis of PCNA. *P < 0.05, ** *P* < 0.01, ***P < 0.001, ****P < 0.0001

To assess the potential of nomilin to inhibit TNBC cell proliferation, we utilized CCK-8 to measure the cell viability of TNBCCs following treatment with different concentrations of nomilin. The results depicted in Fig. 7A and B revealed a concentration-dependent inhibition of TNBCCs proliferation by nomilin. Based on the CCK-8 findings, concentrations of 150 µM and 300 µM were selected for further experimental validation. Curcumin (80 µM) was used as a positive control. Clonogenic assays demonstrated a significantly reduced number of TNBCCs colonies following treatment with 150 µM and 300 µM nomilin compared to the control group (Fig. 7C and E). Given that PCNA is widely used to assess BRCA cell proliferation, we conducted Western blot analysis to evaluate PCNA expression in TNBC cells. As illustrated in Fig. 7F and H, nomilin markedly inhibited the expression of PCNA in TNBCCs. These results collectively indicate that nomilin can dose-dependently inhibit TNBCCs proliferation.

### Nomilin inhibits TNBCCs migration

**Fig. 8:**
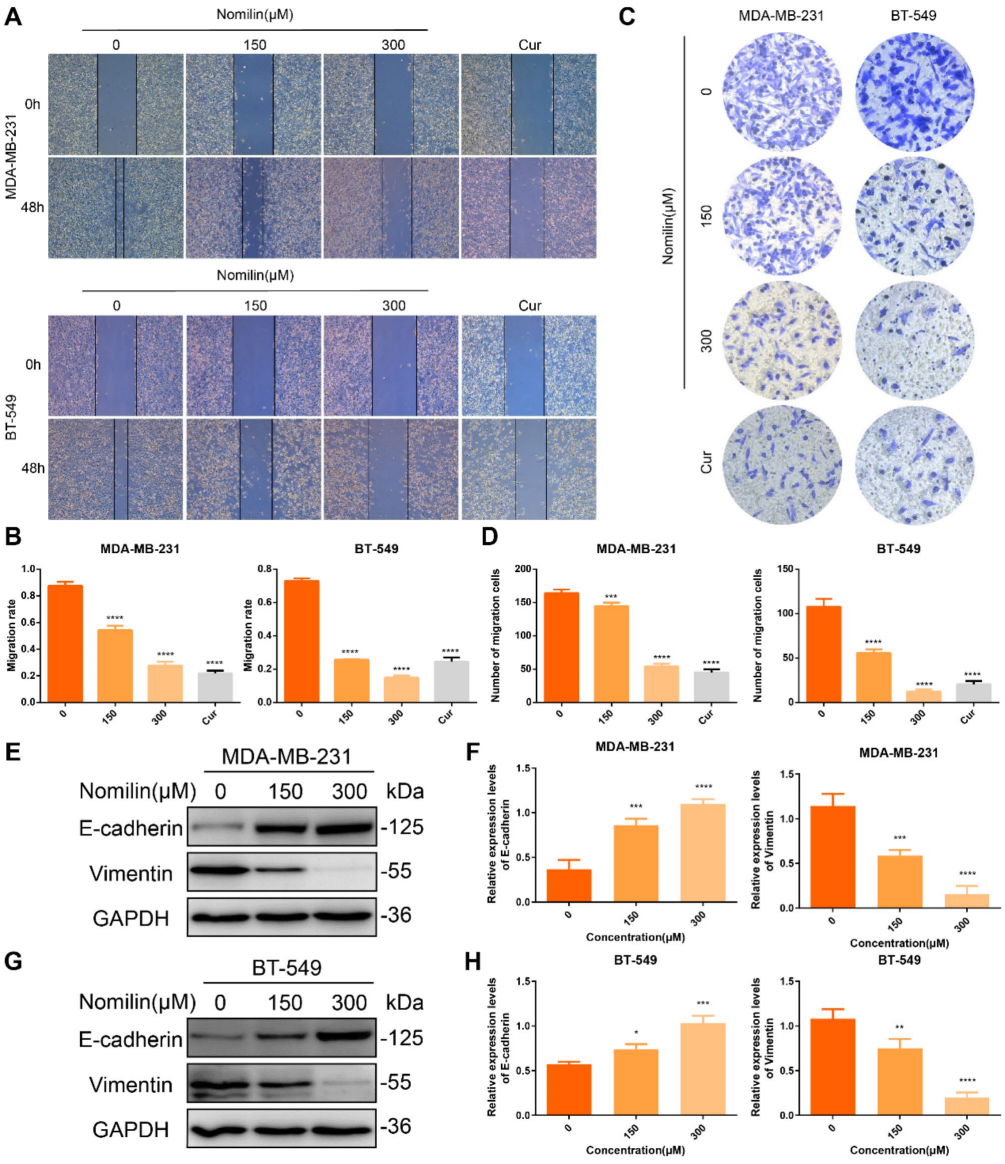
Nomilin inhibits TNBC cell migration. (**A**-**B**) Wound healing assay was used to calculate migration rate. (**C**-**D**) Transwell assay was utilized to evaluate the effect of nomilin treatment on TNBC cells. (**E**-**F**) Western blot analysis of E-cadherin and Vimentin in MDA-MB-231 cells. (**G**-**H**) Western blot analysis of E-cadherin and Vimentin in BT-549 cells. *P < 0.05, ** *P* < 0.01, ***P < 0.001, ****P < 0.0001

We performed wound healing and transwell assays to investigate the potential inhibitory effects of nomilin on the migration of TNBCCs. Figure 8A and D illustrate that untreated MDA-MB-231 and BT-549 cells displayed strong migratory abilities, while nomilin treatment resulted in a notable and concentration-dependent suppression of migration in these cell lines. Furthermore, we sought to investigate whether the observed inhibitory effect of nomilin on TNBC migration was linked to epithelial-mesenchymal transition (EMT). In order to achieve this objective, we evaluated the levels of EMT marker proteins after administering nomilin. Figure 8E and H depicted the WB findings, revealing the significant upregulation of E-cadherin and downregulation of Vimentin due to the prominent effect of nomilin. Collectively, these findings indicate that nomilin can attenuate TNBCCs migration by inhibiting EMT.

### Nomilin induces mitochondria-dependent apoptosis in TNBC cells

**Fig. 9:**
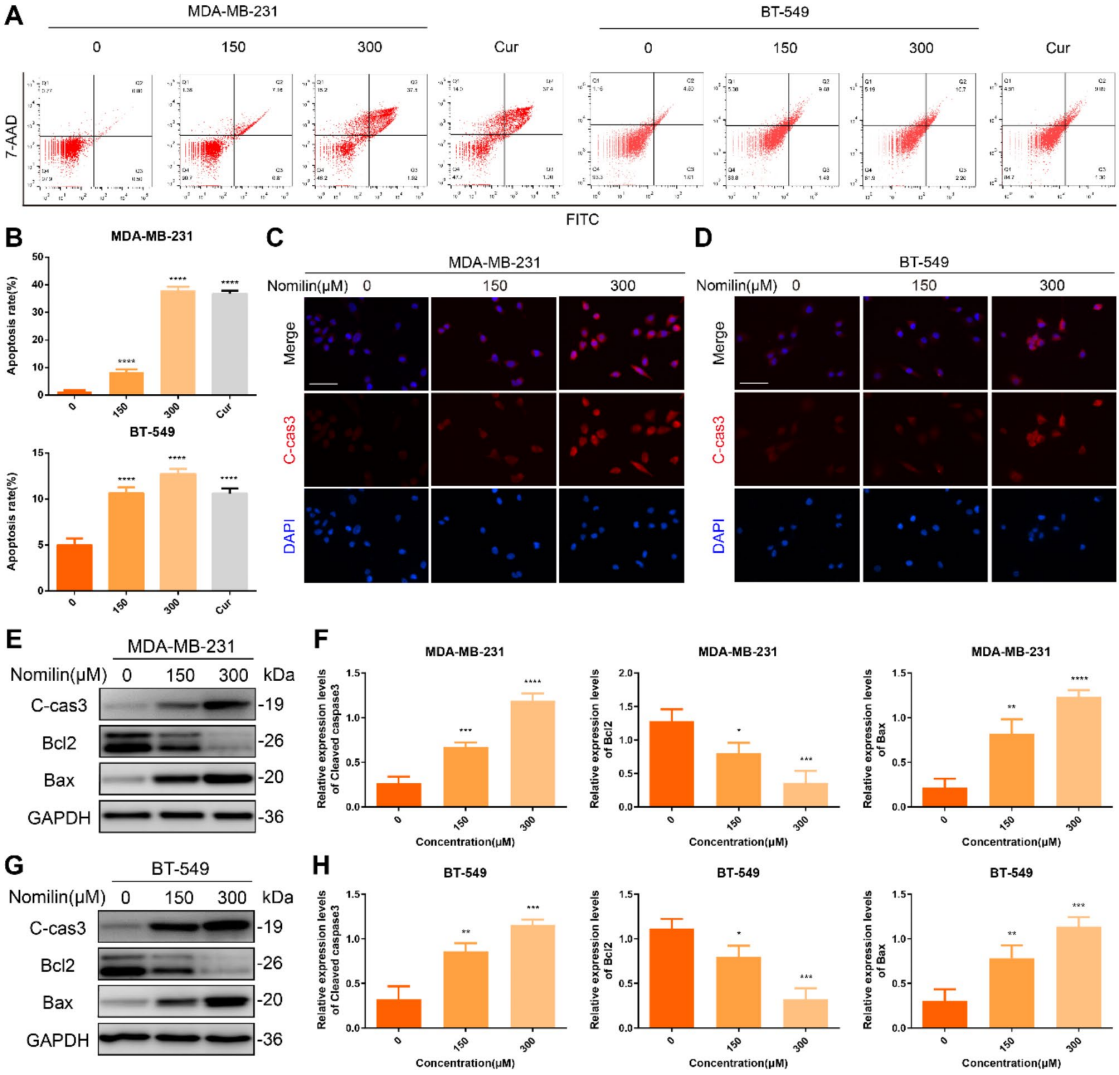
Nomilin induces mitochondria-dependent apoptosis in TNBC cells. (**A**-**B**) Apoptosis of TNBC cells was analysed using flow cytometry. (**C**-**D**) Immunofluorescence analysis of Cleaved-Caspase3. Bar = 50 μm. (**E**-**F**) Western blot analysis of Cleaved-Caspase3, BCL2, and BAX in MDA-MB-231 cells. (**G**-**H**) Western blot analysis of Cleaved-Caspase3, BCL2, and BAX in BT-549 cells. *P < 0.05, ** *P* < 0.01, ***P < 0.001, ****P < 0.0001

Nomilin, associated with its anti-TNBC effects, were enriched in the apoptosis pathway. Furthermore, nomilin was found to exhibit strong binding activity with Caspase3, a key regulator of apoptosis. Based on this, we sought to investigate whether nomilin could induce apoptosis in TNBCCs. The findings depicted in Fig. 9A and B indicate a notable rise in apoptosis in both TNBCCs subsequent to nomilin treatment, with a pronounced increase observed in the percentage of late apoptotic and necrotic cells. The activation of apoptosis is mediated by the cleavage of target cell proteins by the cleaved form of Caspase3. The immunofluorescence analyses showed a notable rise in the brightness of Cleaved-Caspase3 after nomilin treatment (Fig. 9C and D), which aligned with the results obtained from immunoblotting (Fig. 9E and H). Moreover, our previous network pharmacology results indicated that BCL2 was also one of the core targets of nomilin in combating TNBC, with strong binding activity. The findings from WB analysis indicated that the administration of nomilin inhibited the levels of BCL2 while increasing the levels of BAX, indicating that apoptosis induced by nomilin in TNBCCs relies on mitochondria.

#### Effect of nomilin on PI3K/Akt pathway

**Fig. 10:**
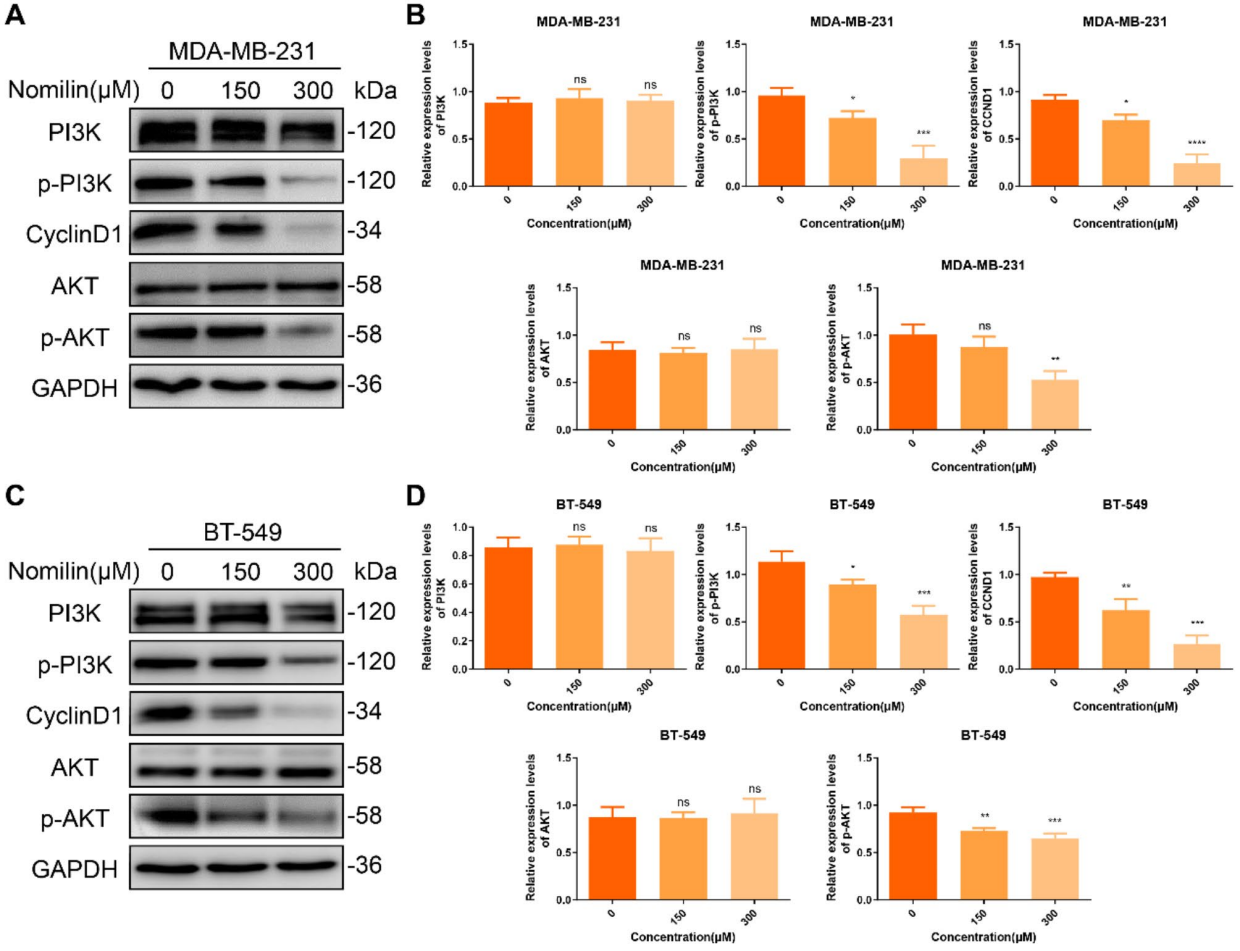
Nomilin inhibits PI3K/Akt/CyclinD1 pathway. (**A**-**B**) Western blot analysis of PI3K, p-PI3K, Akt, p-Akt and CyclinD1 in MDA-MB-231 cells. (**C**-**D**) Western blot analysis of PI3K, p-PI3K, Akt, p-Akt and CyclinD1 in BT-549 cells. *P < 0.05, ** *P* < 0.01, ***P < 0.001, ****P < 0.0001

According to the results of KEGG, it has been indicated that the nomilin may exert an important influence on the anti-TNBC effect by mediating the PI3K/Akt pathway. Subsequently, we conducted a comprehensive examination of nomilin’s role in regulating the PI3K/Akt pathway. Quantitative WB analysis revealed that nomilin reduced the expression of p-PI3K and p-Akt in TNBCCs, while the expression of PI3K and Akt remained unaffected (Fig. 10A and D). Akt has the ability to phosphorylate mTOR, thereby facilitating the upregulation of the cell cycle protein CyclinD1 and promoting the transition from the G1 to the S phase (Gao et al. 2023). Therefore, we assessed the impact of nomilin on CyclinD1 expression. Consistent with the network pharmacology findings, WB results demonstrated that nomilin effectively inhibited the expression of CyclinD1. Collectively, these experimental results substantiate that nomilin exerts its anti-TNBC effect through the PI3K/Akt/CyclinD1 pathway.

#### Nomilin inhibits the growth of TNBC xenograft tumors in nude mice

**Fig. 11:**
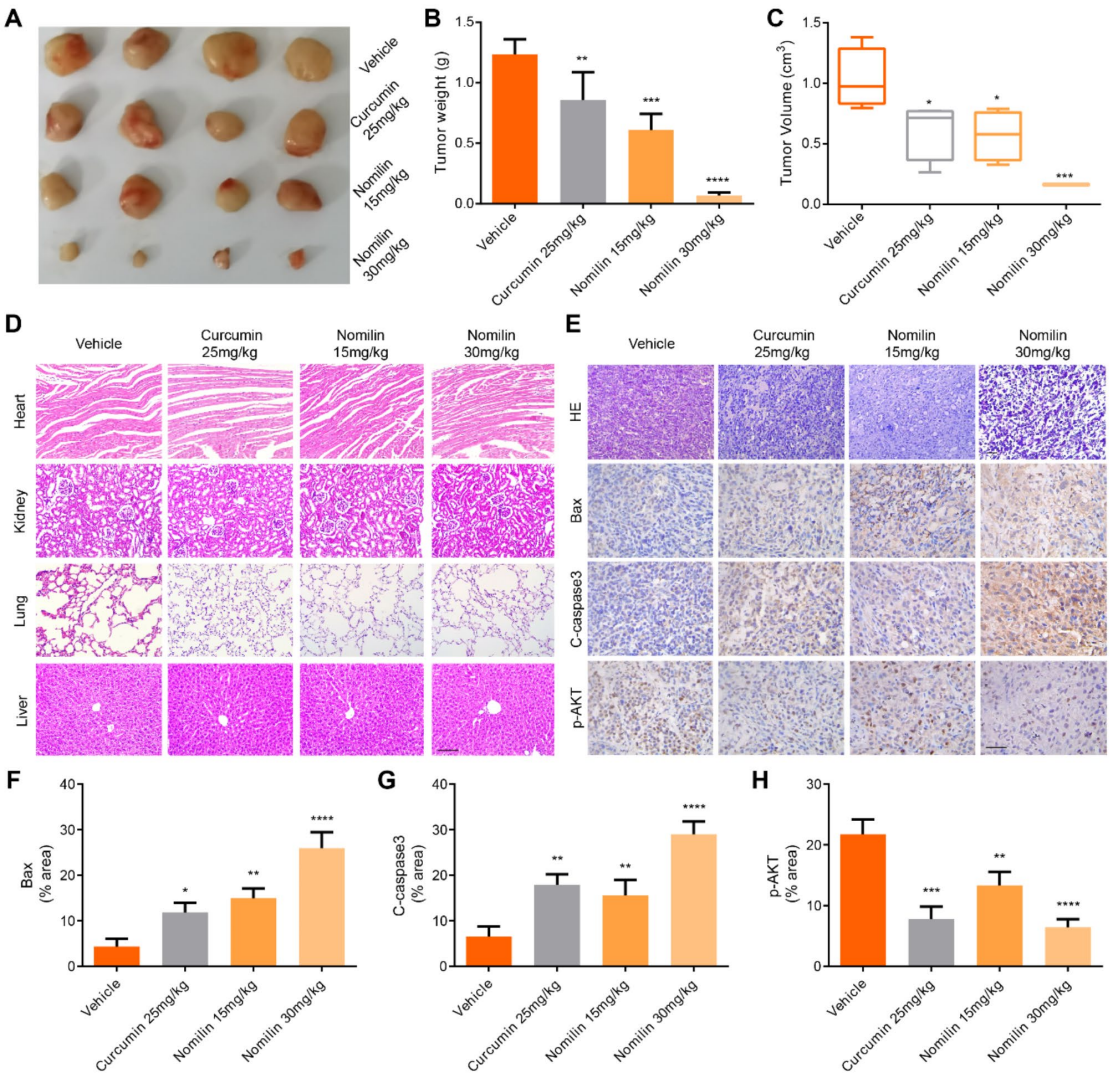
Nomilin inhibits the growth of TNBC xenograft tumors in nude mice. (**A**) Images of resected MDA-MB-231 xenografts. (**B**-**C**) Comparison of the xenograft tumor weight and tumor volume in two groups. (**D**) HE staining of the heart, liver, lungs and kidneys. Bar = 100 μm. (**E**) HE staining of xenograftsand immunohistochemical staining images of Bax, Cleaved-caspase3 and p-Akt. Bar = 50 μm. Quantitative results of immunohistochemical staining of (**F**) Bax, (**G**) Cleaved-caspase3 and (**H**) p-Akt. *P < 0.05, ** *P* < 0.01, ***P < 0.001, ****P < 0.0001

Compared to the control group, the tumor volume and mass of the nomilin-treated nude mice were significantly smaller, as illustrated in Fig. 11A and C. Furthermore, no apparent toxicity of nomilin was observed, as confirmed by HE staining (Fig. 11D). The application of H&E staining on tumor tissue demonstrated a suppression of tumor cell proliferation (Fig. 11E). Additionally, in line with our in vitro findings, nomilin was found to upregulate BAX and Cleaved-Caspase3 expression, indicating that nomilin induces tumor apoptosis through the mitochondria-dependent pathway (Fig. 11E-G). Mechanistically, immunohistochemistry results demonstrated that nomilin reduced the levels of p-Akt (Fig. 11H). Collectively, these research findings indicate that nomilin effectively inhibits tumor growth in vivo and exhibits a favorable safety profile.

## Discussion

TNBC presents a significant challenge in clinical practice due to the absence of specific therapeutic targets. Therefore, it is crucial to create innovative, secure, and efficient alternative treatments for TNBC (Malorni et al. 2012). Several studies have shown that nomilin has a protective effect against a range of cancer types. In pancreatic cancer, neuroblastoma, colon cancer, and estrogen receptor-positive breast cancer, nomilin has shown significant inhibitory effects on tumor cells proliferation (Poulose et al. 2006; Lam and Hasegawa 1989; Kim et al. 2013). However, its effects and mechanisms on different breast cancer subtypes remain unclear, especially in triple-negative breast cancer, which has a poor prognosis. The diverse anti-tumor mechanisms of nomilin make it challenging to elucidate its complex treatment mechanism for TNBC using traditional methods. The innovation and development of network pharmacology have made it possible to uncover the intricate interactions between drugs and their targets, along with their underlying mechanisms at a system level. In this study, we utilized network pharmacology analysis to identify potential targets and pharmacological mechanism of nomilin for the treatment of TNBC. The efficacy of nomilin in binding to the target was ascertained through a comprehensive analysis involving molecular docking, molecular dynamics simulations, and proteome microarray techniques. Through in vitro and in vivo experiments, we have demonstrated that nomilin inhibits TNBC by targeting the PI3K/Akt/CyclinD1 pathway, while also exhibiting a favorable safety profile.

In our initial investigation, we identified 221 potential targets of nomilin against TNBC. Following this, a PPI network was constructed, identifying 8 core targets that were intricately linked to the advancement of TNBC. BCL2, one of the key genes, codes for a protein located in the outer membrane of mitochondria and has a vital function in controlling apoptosis, also known as programmed cell death (Cory et al. 2003). Although BCL2 acts as a pro-survival oncogene in lymphoma (McDonnell and Korsmeyer 1991), it has been found to have a contradictory role as a suppressor gene in different types of tumors, such as BRCA (Zinkel et al. 2006). According to the report, BRCA patients with BCL2-positive tumors exhibit more favorable clinicopathological characteristics, although the underlying reasons for this correlation remain uncertain (Callagy et al. 2006). Some studies speculate that the beneficial prognostic effect may be related to its non-apoptotic function (Lipponen et al. 1995). Our findings suggest that nomilin exhibits strong binding activity with BCL2, and pharmacological inhibition of BCL2 induces apoptosis in TNBC cells. Further research is required to explore the potential functions of BCL2 in BRCA. During the apoptosis process, Caspase-3 acts as the primary enzyme responsible for cutting at the end in the process of apoptosis, generating two active fragments of 17 and 12 kDa (known as Cleaved-Caspase3) to carry out the apoptosis program (Lin et al. 2017; Lakhani et al. 2006). Our study confirmed this process, indicating that nomilin can induce apoptosis by upregulating Cleaved-Caspase3. The CyclinD1 protein serves as a critical cell cycle checkpoint, and it is overexpressed in approximately 50% of primary BRCA patients (Umekita et al. 2002). The prognostic significance of CyclinD1 overexpression remains a topic of debate (Utsumi et al. 2000). Several studies have indicated that the inhibition of CyclinD1 can suppress the proliferation and invasion of BRCA, a result that is consistent with our research (Jiang et al. 2011). The receptor tyrosine kinase EGFR is intricately linked to the initiation, advancement, and outcome of BRCA when exhibiting overexpression or aberrant activation (Li et al. 2022). The complete confirmation of the efficacy of EGFR-targeted therapy in TNBC remains uncertain, necessitating additional research and verification for its clinical application. HSP90AA1, an essential heat shock protein belonging to the HSP90 group, has been linked to enhancing the growth, infiltration, and spread of different cancers, indicating its potential utility as a therapeutic target for cancer management (Liu et al. 2021). The KRAS protein, belonging to the RAS superfamily of small GTPases, has been strongly associated with decreased survival rates in BRCA patients when expressed at elevated levels (Hwang et al. 2019). PARP1, an integral part of the polymerase group, has a crucial function in the nucleus for the mending of single-strand fractures during the process of DNA damage repair (Zuo et al. 2020). For BRCA mutation-related breast cancer, PARP1 inhibitors such as Olaparib are widely used in clinical treatment (Noordermeer and Attikum 2019). TNF, or tumor necrosis factor, is an important pro-inflammatory cytokine implicated in the occurrence and progression of human cancer (Yang et al. 2011). The persistent secretion of TNF-α by both tumor cells and the local microenvironment has the potential to enhance the growth and spread of BRCA (Cruceriu et al. 2020). In summary, these 8 core targets play crucial roles in the onset, development, and prognosis of TNBC, and they have the potential to become targets for nomilin against TNBC. Computer-assisted molecular docking, molecular dynamics simulations and proteome microarray analysis further revealed that these eight core targets are direct targets of nomilin. Additional research is required to clarify the roles of these essential targets in TNBC and to investigate the mechanism of interaction between nomilin and these targets.

The KEGG results revealed that the eight core targets were predominantly enriched in the PI3K/Akt, AGE-RAGE, apoptosis, and microRNAs in cancer signaling pathways, the majority of which are linked to cancer. EGFR activation, KRAS mutation, and TNF-α binding to the receptor can trigger PI3K activation (Freudlsperger et al. 2011; Mortazavi et al. 2022; Gonzalez Caldito 2023). HSP90AA1 is essential for the proper folding and stabilization of PI3K and Akt, ensuring normal pathway function (Zhang et al. 2023). Additionally, PARP1 regulates PI3K and Akt through PARylation, modifying their phosphorylation status, stability, and overall activity (Dias et al. 2021). Upon activation, the PI3K/Akt pathway plays a crucial role in the regulation of cell survival by directly phosphorylating Bcl2 and caspase3 (Hers et al. 2011). Akt activation also induces mTOR phosphorylation and upregulates the expression of cyclin D1 (Gao et al. 2023). These findings indicate that there is a significant relationship between eight core targets and the PI3K/Akt pathway. The PI3K/Akt pathway has been found to be abnormally activated in different types of tumors, resulting in an exaggerated reaction of tumor cells to growth factors and facilitating the proliferation and spread of tumors (Rhun et al. 2017; Xu et al. 2015). BRCA is highly characterized by the disturbance of the PI3K/Akt pathway, with changes detected in 70% of instances (Hinz and Jucker 2019). This aberrant activation is also common in TNBC, particularly in the luminal androgen receptor (LAR) subtype as per the Fudan classification (Lawrence et al. 2014). Consequently, our investigation has concentrated on the PI3K/Akt pathway to clarify the mechanism of nomilin’s impact on TNBC.

To further confirm the findings of the network pharmacology analysis, we carried out experiments both in vivo and in vitro. Nomilin effectively suppressed the growth of TNBCCs, as evidenced by Western blot experiments, CCK-8, clonal proliferation, and PCNA assays. TNBC, which is strongly linked to basal-like breast cancer, demonstrates a significant expression of the EMT gene signature (Liu et al. 2022). EMT is closely linked to tumor invasion, metastasis, and recurrence (Dave et al. 2012), and our findings suggest that nomilin can suppress the EMT process to inhibit TNBC migration. The enrichment analysis findings revealed that the apoptosis pathway showed significant enrichment in eight core targets. The study shows that nomilin can trigger cell death in melanoma and pancreatic cancer cells (Chidambara Murthy et al. 2021; Pratheeshkumar et al. 2012). Nevertheless, the assessment of nomilin’s capacity to trigger apoptosis in TNBC cells has yet to be carried out. The results of our study show that nomilin can effectively trigger apoptosis in TNBC cells in a manner that depends on the dosage, by activating the mitochondrial pathway. Further examinations indicate that the phosphorylation of PI3K and Akt is considerably suppressed compared to the control group, while the overall quantities of PI3K and Akt remain unchanged. Moreover, based on our previous results, we have identified CyclinD1 as one of the primary targets of nomilin. Previous study has demonstrated that Akt facilitates mTOR phosphorylation, consequently upregulating the expression of CyclinD1 (Gao et al. 2023). Our data unequivocally indicate that nomilin effectively suppresses CyclinD1 expression. Furthermore, consistent outcomes were observed in xenograft tumor experiments. Therefore, the experimental results mentioned above confirm that the PI3K/Akt/CyclinD1 pathway mediates the anti-TNBC effect of nomilin.

This study also has certain limitations. Our research is mainly based on network pharmacology and in vitro and in vivo experiments, and there is a lack of clinical trials to verify the reliability of nomilin. At the same time, we did not conduct basic experimental verification on all core proteins, which requires further research to explore the mechanism of action of nomilin.

## Conclusion

The study utilized network pharmacology to systematically explore the potential targets and mechanisms of nomilin against TNBC, and these findings were further validated through in vivo and in vitro experiments. Our results illustrate that nomilin inhibits TNBC progression by modulating the PI3K/Akt/CyclinD1 pathway in TNBC, underscoring nomilin as a promising candidate for TNBC treatment.

## Electronic supplementary material

Below is the link to the electronic supplementary material.

Supplementary Material 1

Supplementary Material 2

Supplementary Material 3

Supplementary Material 4

Supplementary Material 5

## Data Availability

The datasets supporting the conclusions of this article are included within the article.
