# Correction: Integrating network pharmacology, molecular docking and experimental verification to explore the therapeutic effect and potential mechanism of nomilin against triple-negative breast cancer

**DOI:** 10.1186/s10020-025-01188-4

**Published:** 2025-04-16

**Authors:** Zhixuan Wu, Haoyi Xiang, Xiaowu Wang, Rongrong Zhang, Yangyang Guo, Liangchen Qu, Jingyao Zhou, Yanyi Xiao

**Affiliations:** 1 The Dingli Clinical College of Wenzhou Medical University, Wenzhou, Zhejiang Province 325000 China; 2 Zhejiang Key Laboratory of Intelligent Cancer Biomarker Discovery and Translation, First Affiliated Hospital, Wenzhou Medical University, Wenzhou, Zhejiang Province 325035 China; 3 Zhejiang University School of Medicine, Hangzhou, Zhejiang Province 310016 China; 4 Department of Colorectal Surgery, Sir Run Run Shaw Hospital of Zhejiang University, Hangzhou, Zhejiang Province 310016 China; 5 Department of Burns and Skin Repair Surgery, The Third Affiliated Hospital of Wenzhou Medical University, Ruian, 325200 China; 6 Emergency Department, Taizhou Hospital of Zhejiang Province Affiliated to Wenzhou Medical University, Taizhou, 318000 China; 7 Pharmacy Department, Taizhou Central Hospital, Taizhou, Zhejiang Province 318000 China; 8 Department of Thyroid and Breast Surgery, Wenzhou Central Hospital, The Second Affiliated Hospital of Shanghai University, Wenzhou, Zhejiang Province 325000 China

**Molecular Medicine (2024) 30:166**

10.1186/s10020-024-00928-2

In this article (Wu et al. 2024) the affiliation details for the author Zhixuan Wu were incorrectly given as ‘The Dingli Clinical College of Wenzhou Medical University, Wenzhou, Zhejiang Province 325000, China’ and ‘Zhejiang Key Laboratory of Intelligent Cancer Biomarker Discovery and Translation, First Affiliated Hospital, Wenzhou Medical University, Wenzhou, Zhejiang Province 325035, China’ but should have been ‘Zhejiang Key Laboratory of Intelligent Cancer Biomarker Discovery and Translation, First Affiliated Hospital, Wenzhou Medical University, Wenzhou, Zhejiang Province 325035, China.’

The original article has been corrected.
