# Supplementary figures and images for: Integrating network pharmacology, molecular docking and experimental verification to explore the therapeutic effect and potential mechanism of nomilin against triple-negative breast cancer
Source: Mol Med. 2024 Sep 28;30:166. doi: 10.1186/s10020-024-00928-2 (PMC11439318; doi:10.1186/s10020-024-00928-2)

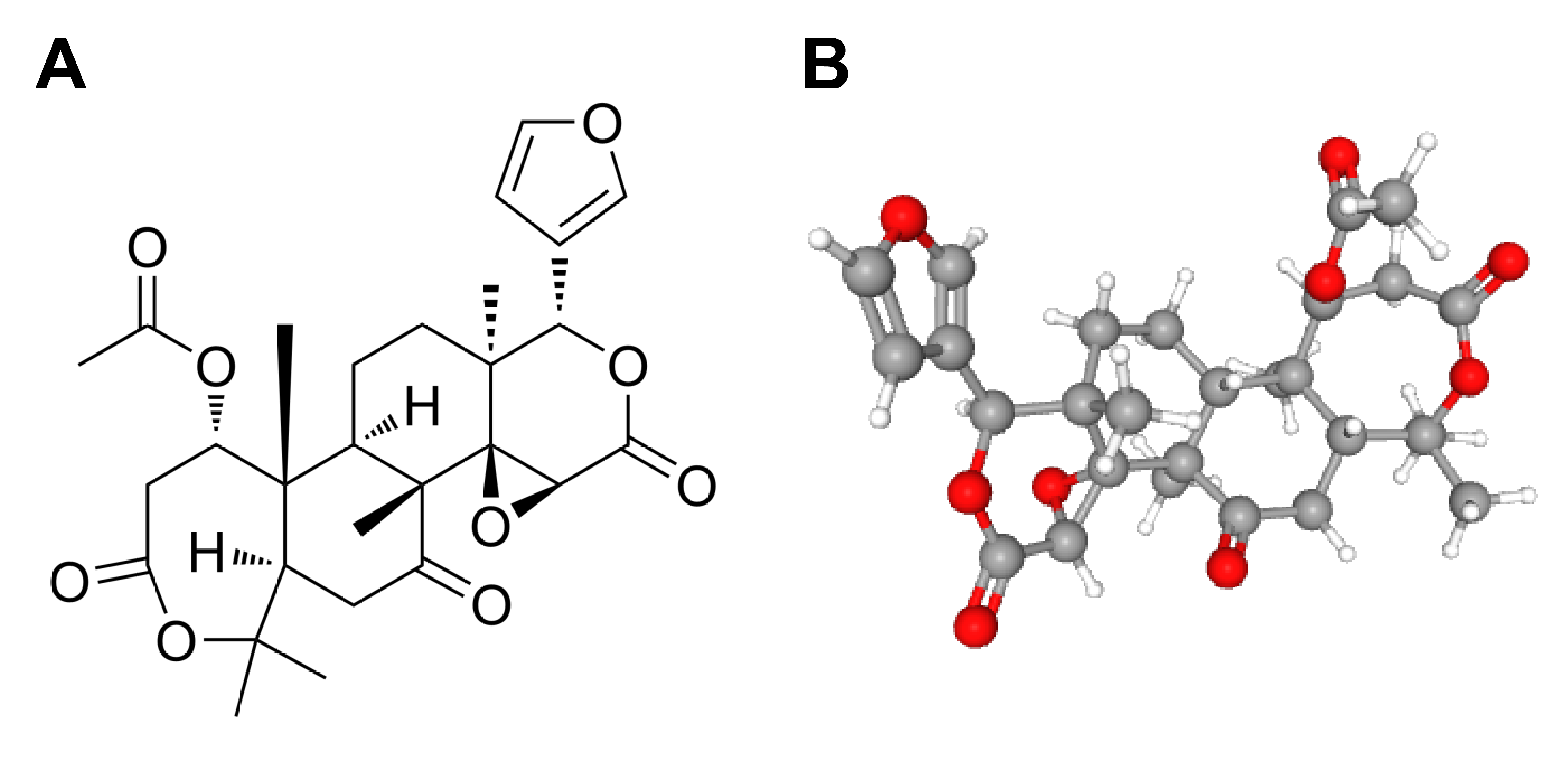

Supplement: Supplementary file 1 — Supplementary Material 1 [file 10020_2024_928_MOESM1_ESM.tif]

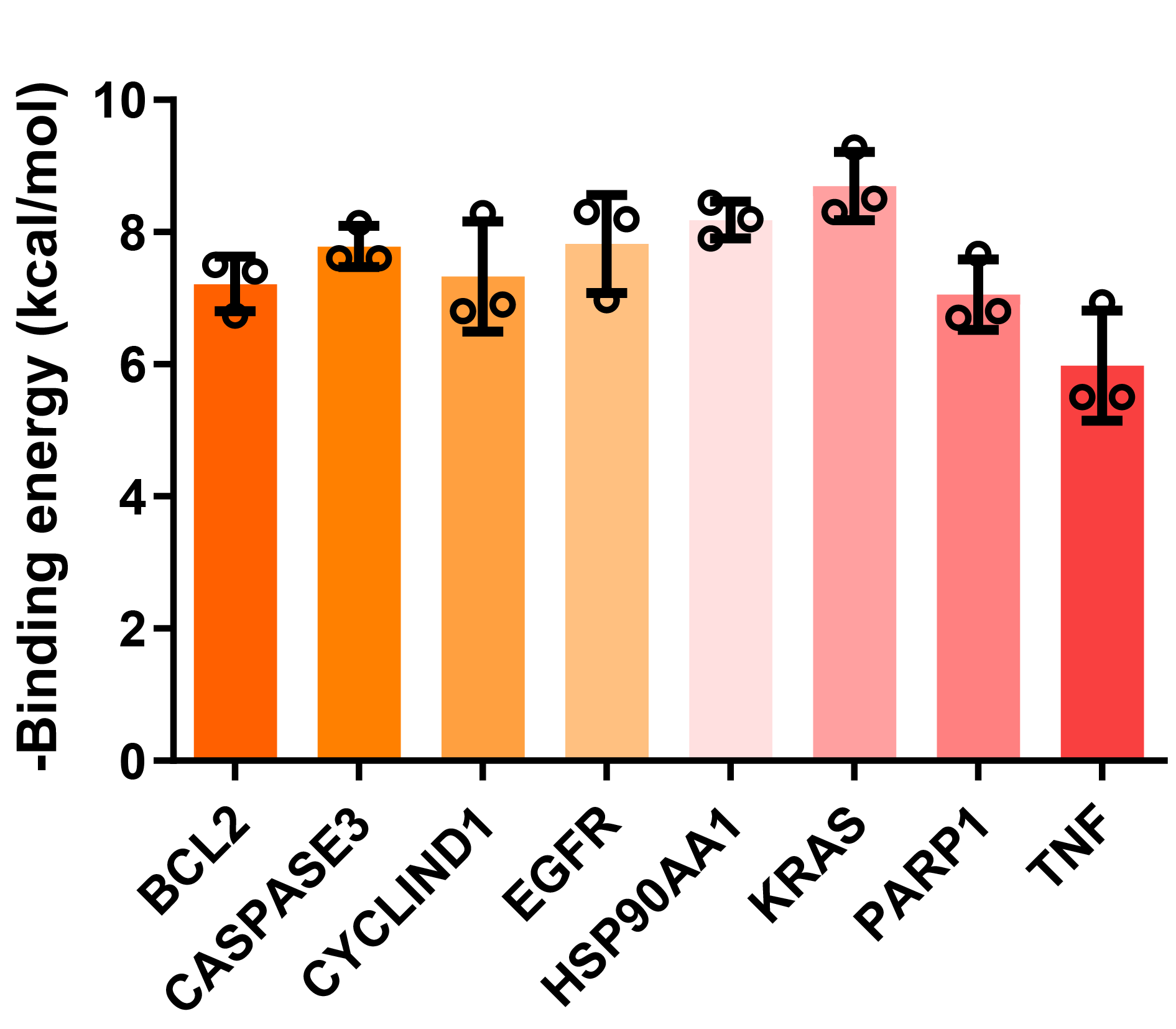

Supplement: Supplementary file 2 — Supplementary Material 2 [file 10020_2024_928_MOESM2_ESM.tif]

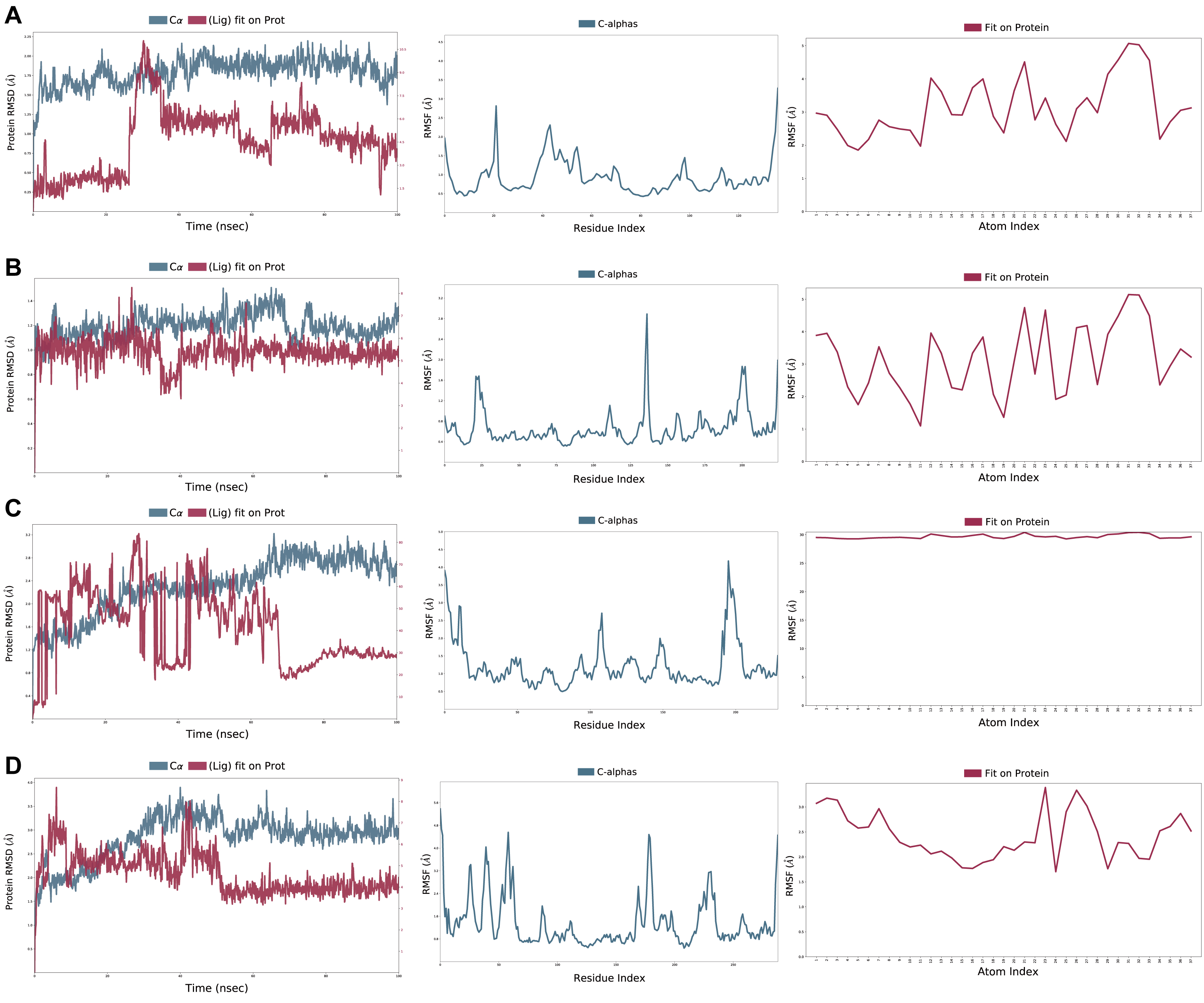

Supplement: Supplementary file 3 — Supplementary Material 3 [file 10020_2024_928_MOESM3_ESM.jpg]

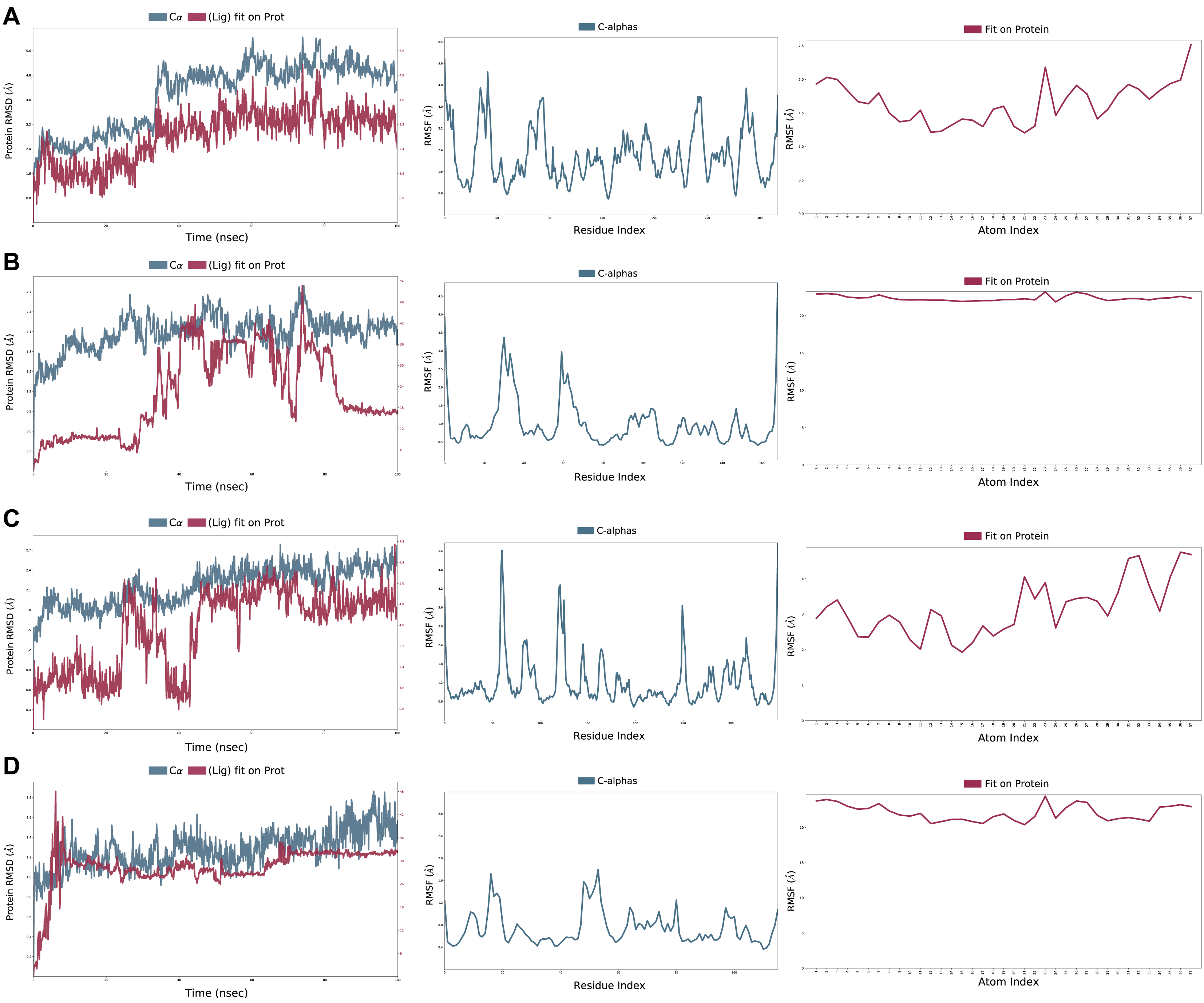

Supplement: Supplementary file 4 — Supplementary Material 4 [file 10020_2024_928_MOESM4_ESM.jpg]
